# Selection of dilution material for non-iodinated iodine as an oral contrast agent for esophageal cancer: a preliminary clinical trial

**DOI:** 10.1007/s11604-022-01299-6

**Published:** 2022-07-20

**Authors:** Xia Xu, Zhifeng Wu, Na Zhang, Ziquan Guo

**Affiliations:** Department of Radiology, Shanxi Bethune Hospital, Taiyuan, Shanxi China

**Keywords:** Esophageal cancer, Esophagus, Computed tomography, Contrast agent

## Abstract

**Purpose:**

To investigate the filling state of the esophagus using different oral contrast agents for the diagnosis of esophageal cancer by computed tomography (CT).

**Materials and methods:**

This preliminary clinical trial enrolled patients with suspected esophageal carcinoma and admitted from January 2015 to January 2018. The patients were randomized into the yogurt (mixed with ioversol), lotus root powder (mixed with ioversol), gas-producing powder, and control (pure iodine water) groups. Chest CT examinations were performed. The degree of esophageal filling and the detection of esophageal lesions were compared.

**Results:**

Finally, 136 participants were enrolled (*n* = 34/group). There were no significant differences in esophageal filling degree among the yogurt, lotus root powder, and gas-producing powder groups (*P* = 0.093). There were 30/3/1 and 30/3/1 confirmed/false-negative/false-positive diagnoses in the yogurt and lotus powder groups, respectively, compared with 28/5/1 and 25/8/1 in the gas-producing powder and control groups, respectively. The concordance rates were the highest for the yogurt (88.2%, with 91.7% specificity and 86.4% sensitivity) and lotus root powder groups (88.2%, with 92.3% specificity and 85.7% sensitivity) and the lowest for the control group (73.5%, with 90.0% specificity and 66.7% sensitivity).

**Conclusion:**

Yogurt mixed with ioversol could fill and expand the esophagus with minimal preparation, displaying the structure of the esophageal lumen and wall thickness. This mixture might be used as a positive contrast agent for esophageal CT. Similar results were observed for the lotus root powder mixed with ioversol, but its preparation was more arduous.

## Introduction

Esophageal carcinoma is the eighth most common malignant tumor worldwide [1, 2]. Squamous cell carcinoma comprises 90% of all esophageal cancers [3–6]. Squamous cell carcinoma is reported to be more common in Eastern Europe and Asia, while adenocarcinoma is reported to be more common in North America and Western Europe [4–6]. Most patients affected with esophageal cancer are > 50 years old, and both histologic subtypes are more common in men [3–5]. The most likely risk factors for esophageal cancer include tobacco use, excessive alcohol use, a history of gastroesophageal reflux disease (GERD), head and neck squamous cell carcinoma, and Barrett esophagus [4–6]. Most tumors are diagnosed along with regional or distant metastasis, which decreases the overall 5-year survival from 39% for localized disease to 4% for distant metastases [3–5].

The American Joint Committee on Cancer (AJCC) TNM system is routinely used to stage esophageal cancer [5, 7]. In addition, a variety of imaging modalities, including ultrasound, endoscopic ultrasound (EUS), computed tomography (CT), magnetic resonance imaging (MRI), and positron emission tomography (PET)/CT, can be used for staging and monitoring treatment efficacy in patients with esophageal cancer [5, 8, 9]. Most patients undergo CT examinations to assess tumor invasion of adjacent structures and tumor metastasis [5, 8–10]. On the other hand, in most comparative studies, the accuracy of CT in evaluating the T stage is lower than that of EUS [11, 12].

When using CT for esophageal cancer, the expansion of the lumen is an important factor because the collapsed lumen may mask the disease and produce false positives due to muscle contraction [13–15]. Therefore, filling the esophageal lumen with a contrast agent could help prevent artifacts and provide a better view.

Therefore, this preliminary clinical trial aimed to investigate the filling state of the esophagus with different oral contrast agents for the diagnosis of esophageal cancer by CT. The results could provide better methods to display the esophagus and provide more evidence for management.

## Materials and methods

### Study design and participants

This preliminary clinical trial enrolled patients with suspected esophageal carcinoma and admitted to the Department of Radiology of our Hospital from January 2015 to January 2018. The clinical case system was queried each morning, and the outpatient doctor was informed of the potentially eligible patients. This study was approved by the Ethics Committee of our Hospital. All methods were performed in accordance with the relevant guidelines. All patients signed the study informed consent form before any examination.

The inclusion criteria were (1) suspected esophageal carcinoma according to the main clinical manifestations (including choking after eating, retrosternal pain, progressive dysphagia, back pain, and emaciation) [4–6], (2) volunteered to participate in the study, and (3) underwent gastroscopy or pathological examination within 2 weeks after the CT examination. The exclusion criteria were (1) unable to cooperate with the examinations due to consciousness disorder or severe dysphagia, (2) allergy to dairy products, lotus, or contrast agents, (3) lactose intolerance, or (4) already diagnosed with esophageal carcinoma and underwent radiotherapy or chemotherapy or postoperative check-up.

Using the random number table method, the participants were randomly divided into the yogurt group, lotus root powder group, gas-producing powder group, and control (pure iodine water) group. All patients underwent chest CT scans.

### Contrast agent preparation

The thick Yili AnMushi 205 g packaged pasteurized flavored yogurt (Inner Mongolia Yili Industrial Group Co., Ltd., China) was selected for this study. It was mixed with 6 ml of ioversol 33.9 g/50 ml (Hengrui Medicine Co. Ltd., Jiangsu, China). Commercial Paradise sugar-free instant pure lotus root powder (10 g, Hangzhou Paradise Food Co., Ltd., China) was mixed with 5 ml of cold purified water, poured into 70 ml of boiled water above 95 °C, stirring quickly to obtain a viscous paste, and 5 ml of ioversol (33.9 g/50 ml, Hengrui Medicine Co. Ltd., Jiangsu, China) was added and mixed. A gas-producing powder (Qingdao Dongfang Chemical Co., Ltd., China) was selected, with 3 g as one dose.

### Oral contrast agent administration method

Before imaging, the staff enquired about two aspects: (1) the food intake of the participant to know whether the contrast agents studied here could be taken (specifically, if the participant could not swallow liquid foods, he/she was not included in this study to prevent aspiration or other complications caused by vomiting), and (2) determine whether there was an allergy to any of the compounds used in this study, including iodine and milk.

In this study, a non-iodinated iodine contrast agent was used instead of barium sulfate mainly because barium sulfate is a particularly viscous and non-absorbable contrast agent, and critical aspiration pulmonary edema has not been reported like with an iodinated iodine contrast agent.

The participants in the yogurt and lotus root powder groups took orally about 30–50 ml of the prepared contrast agent before scanning and were asked to take a big mouthful of the agent (about 20–30 ml). After the positioning image was ready, the participants were asked to swallow it immediately before starting the scan. Starting scanning, the participants in the gas-producing powder group swallowed 3 g of powder and an appropriate amount of warm water during scanning. The participants in the control group swallowed 250 ml of iodine solution (containing 5 ml of ioversol) before lying on the CT table. For swallowing the contrast agent, the patients took the agent in their mouth in the sitting position, took the supine position, and swallowed it when the technician gave the signal. No intravenous contrast was used.

### CT examination and evaluation

A Somatom Definition Flash dual-source CT scanner (Siemens, Erlangen, Germany) was used for scanning the participants in the supine position. The scanning range was from the thoracic entrance to the gastric fundus level. The scan time was 3 s, the CT tube voltage was 120 kV, and the reference tube current-time product was 180 mAs. Real-time dynamic exposure dose adjustment was enabled, regulating the collimation to 0.6 mm × 128, the rotation time to 0.5 s/turn, the pitch factor to 0.9, the slice thickness to 5 mm, and the reconstructed image slice thickness to 1 mm. The images were transmitted to the workstation for analysis and reconstruction.

Two CT radiologists with more than 5 years of CT diagnosis experience analyzed the images. The wall of the esophagus is concentric and about 4 mm thick. The images were verified for the presence of a mass in the esophageal lumen, esophageal stenosis, invasion of surrounding tissues, and distant metastasis [5, 14, 16]. If the radiologists had any disagreement on the diagnosis result, they reported to the general practitioner for a consultation. The unified conclusion was used as the final CT diagnosis result. The subjective scoring criteria for the esophageal filling degree were 1 point for poor esophageal filling, indistinguishable lumens, and unable to meet the diagnostic needs, 2 points for normal esophageal filling but incomplete dilation with lumen surface shrinkage, 3 points for good esophageal filling, and well-dilated esophagus, and 4 points for excellent esophageal filling and fully dilated esophagus [17]. The agreement between the two radiologists was evaluated using the *κ* statistic: *κ* > 0.75 was considered good consistency, *κ* of 0.40–0.74 indicated moderate consistency, and *κ* < 0.40 indicated poor consistency.

### Gastroscopy or postoperative pathology

Within 2 weeks after CT, all participants underwent gastroscopy or surgery. All the lesions that appeared suspicious to the gastroenterologists were biopsied, and a pathologist routinely examined the specimens.

### Statistical analysis

All analyses were carried out using SPSS 22.0 (IBM, Armonk, NY, USA). Continuous variables were presented as median (range) and analyzed using the Kruskal–Wallis test. Categorical data were presented as n (%) and analyzed using the chi-square test or Fisher’s exact test, as appropriate. Two-sided *P*-values < 0.05 were considered statistically significant.

## Results

### Characteristics of the participants

**Fig. 1:**
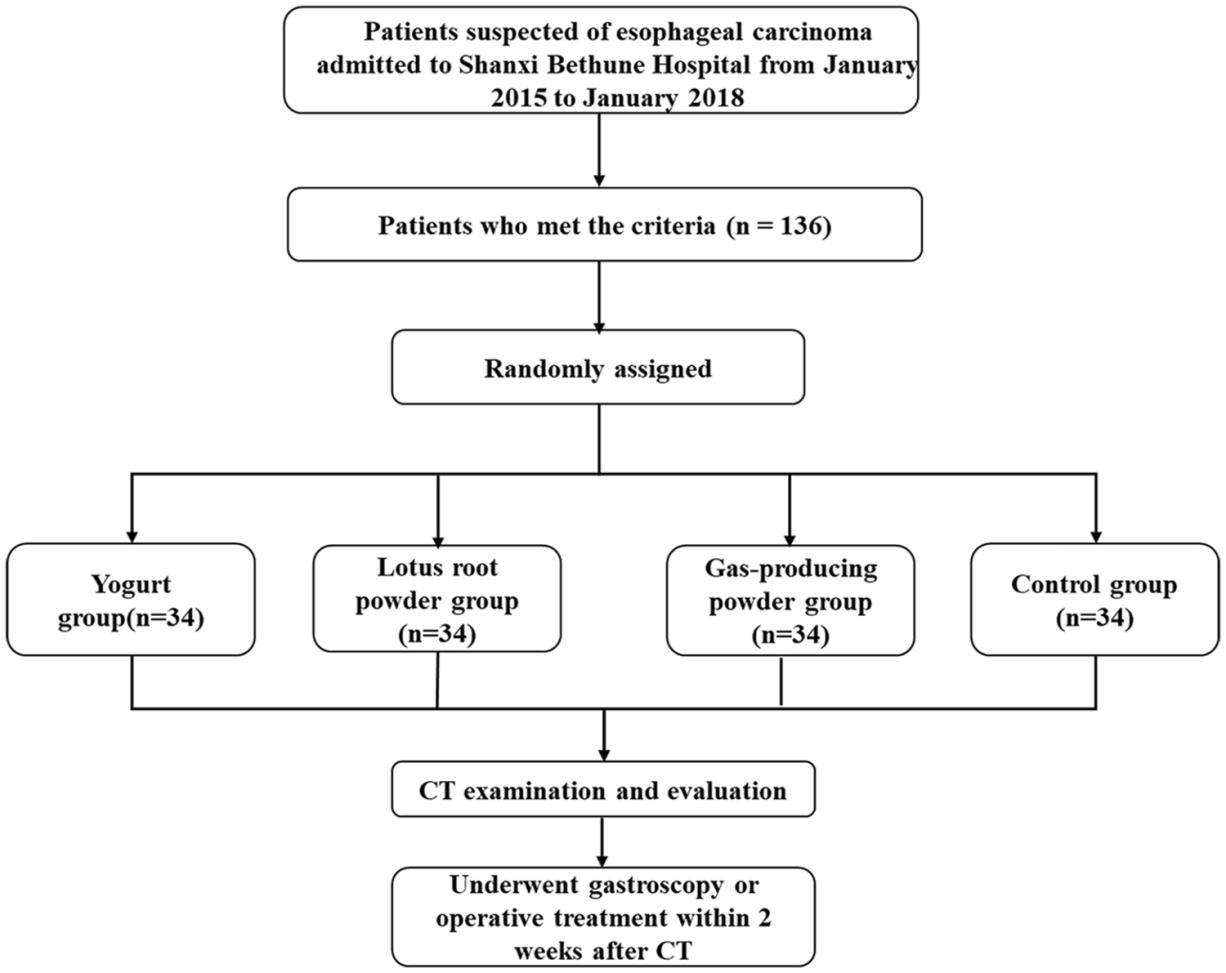
Flow chart of patient enrollment

Figure 1 presents the participant flowchart. All 136 participants completed the CT examination using the assigned oral contrast agent. There were 19 males and 15 females in the yogurt group; they were 41–73 years old. There were 20 males and 14 females in the lotus root powder group; they were 39–75 years. There were 18 males and 16 females in the gas-producing powder group; they were 39–83 years old. In the control group, there were 19 males and 15 females; they were 43–79 years old. The characteristics of the four groups were comparable (all *P* > 0.05) (Table 1). The diagnosis of all 136 patients was confirmed by gastroscopy or pathology. Among them, 86 participants were diagnosed with esophageal cancer (77 with squamous carcinoma and 9 with adenocarcinoma), and 50 patients were suspected cases.

**Table 1:** Baseline characteristics of the patients

Characteristics	Yogurt (*n* = 34)	Lotus root powder (*n* = 34)	Gas-producing powder (*n* = 34)	Control (*n* = 34)	*P*
Age, years, median (range)	52 (41–73)	49 (39–75)	57 (39–83)	53 (43–79)	0.221
Sex (male/female)	19/15	20/14	18/16	19/15	0.971
Symptoms, *n* (%)					
A lump in the throat after eating	29 (85.3)	31 (91.2)	31 (91.2)	32 (94.1)	0.656
Pain behind the sternum	18 (52.9)	16 (47.1)	13 (38.2)	15 (44.1)	0.673
Progressive difficulty swallowing	7 (20.6)	9 (26.5)	4 (11.8)	11 (32.4)	0.215
Emaciation	10 (29.4)	7 (20.6)	9 (26.5)	5 (14.7)	0.482
Pathologically confirmed, *n* (%)	22 (64.7)	21 (61.8)	19 (55.9)	24 (70.6)	0.649

### CT evaluation of esophageal filling degree with different oral contrast agents

**Fig. 2:**
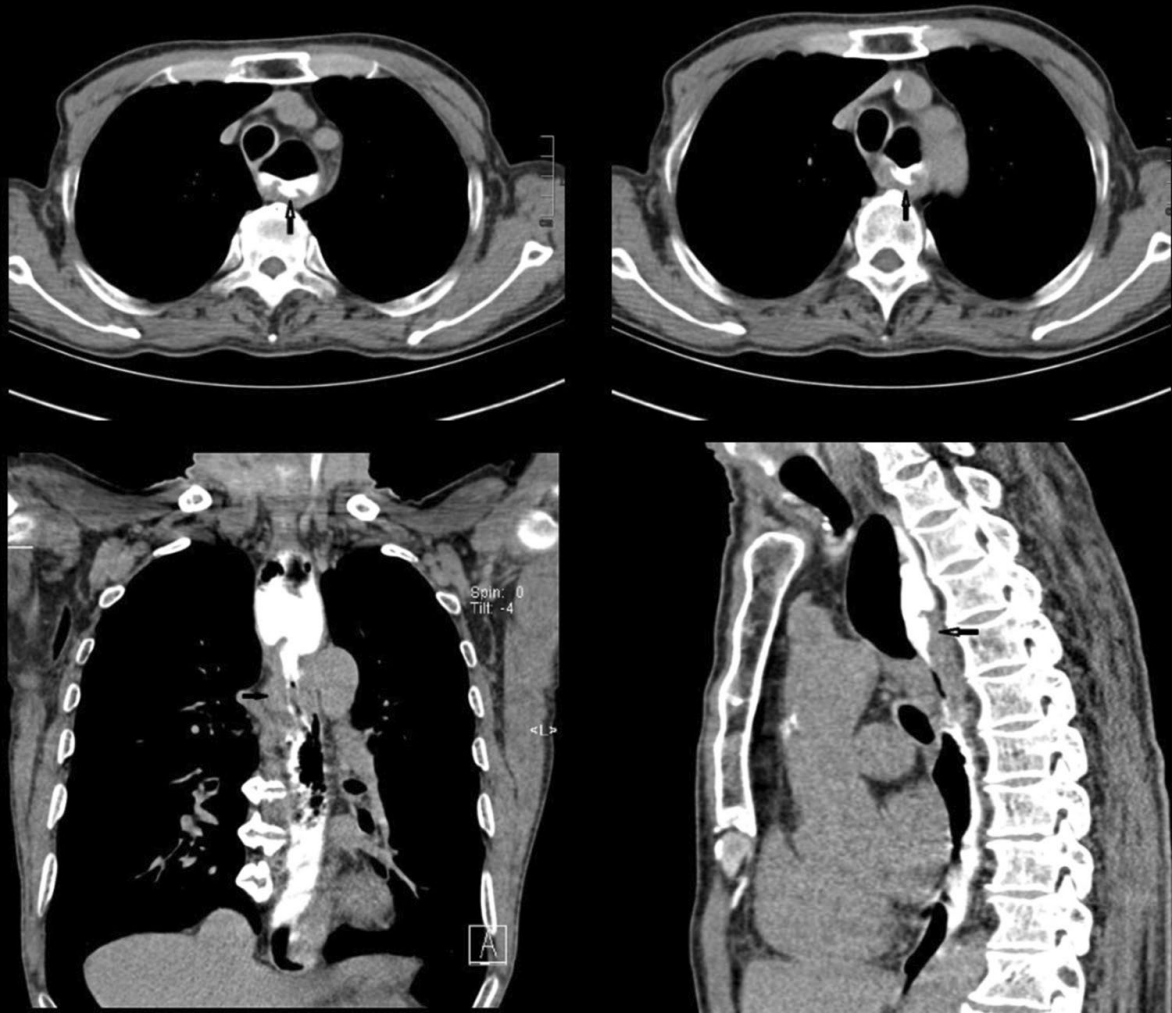
Computed tomography (CT) images of the yogurt group. (**A**, **B**) Axial CT images showing thickening of the esophageal wall with ulceration. **C** CT coronal image, showing significant thickening of the esophageal wall and narrowing of the lumen. **D** CT sagittal image showing esophageal wall thickening with ulceration

**Fig. 3:**
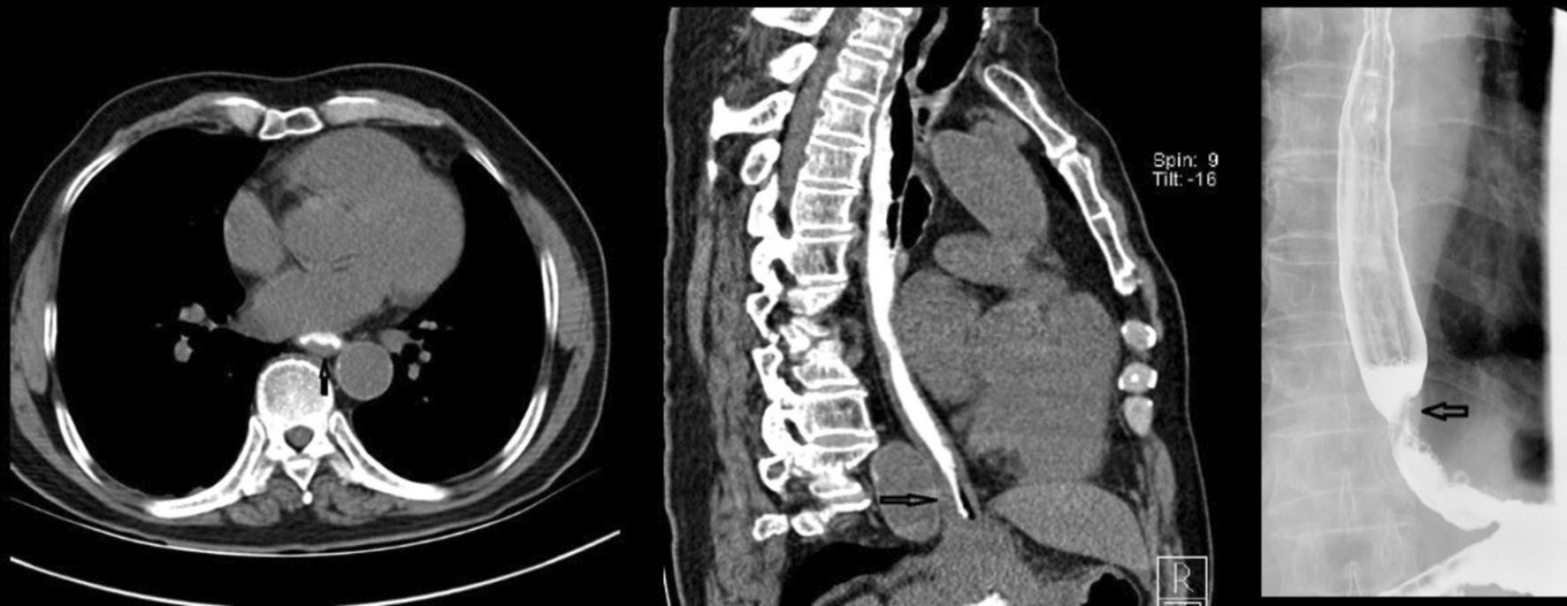
Computed tomography (CT) image of the esophagus filled with lotus root powder. **A** Thickening of the wall of the lower segment of the esophagus, especially the thickening of the posterior wall. **B** CT sagittal image of the esophagus filled with lotus root powder, showing thickening of the lower esophagus wall and the back wall. **C** Esophageal barium meal contrast image, showing lumen stenosis in the lower segment of the esophagus, which was consistent with the CT images

**Fig. 4:**
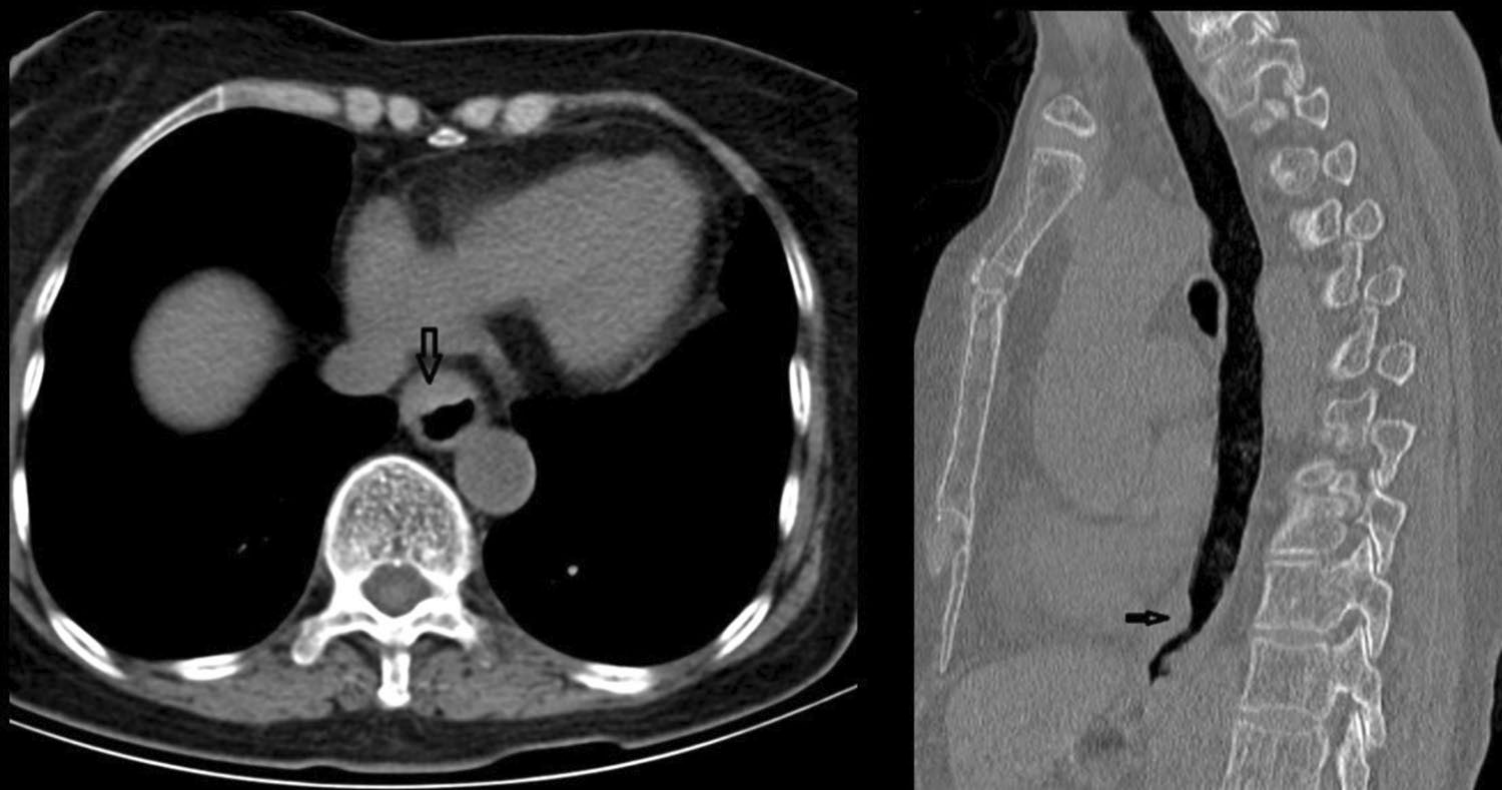
Computed tomography (CT) image of a filling esophagus with gas-producing powder. **A** CT axial image of esophageal filling with gas powder, showing a thickening of the esophageal wall. **B** Sagittal view of the filling gas powder of the esophagus, showing thickening of the esophageal wall and narrowing of the lumen

**Fig. 5:**
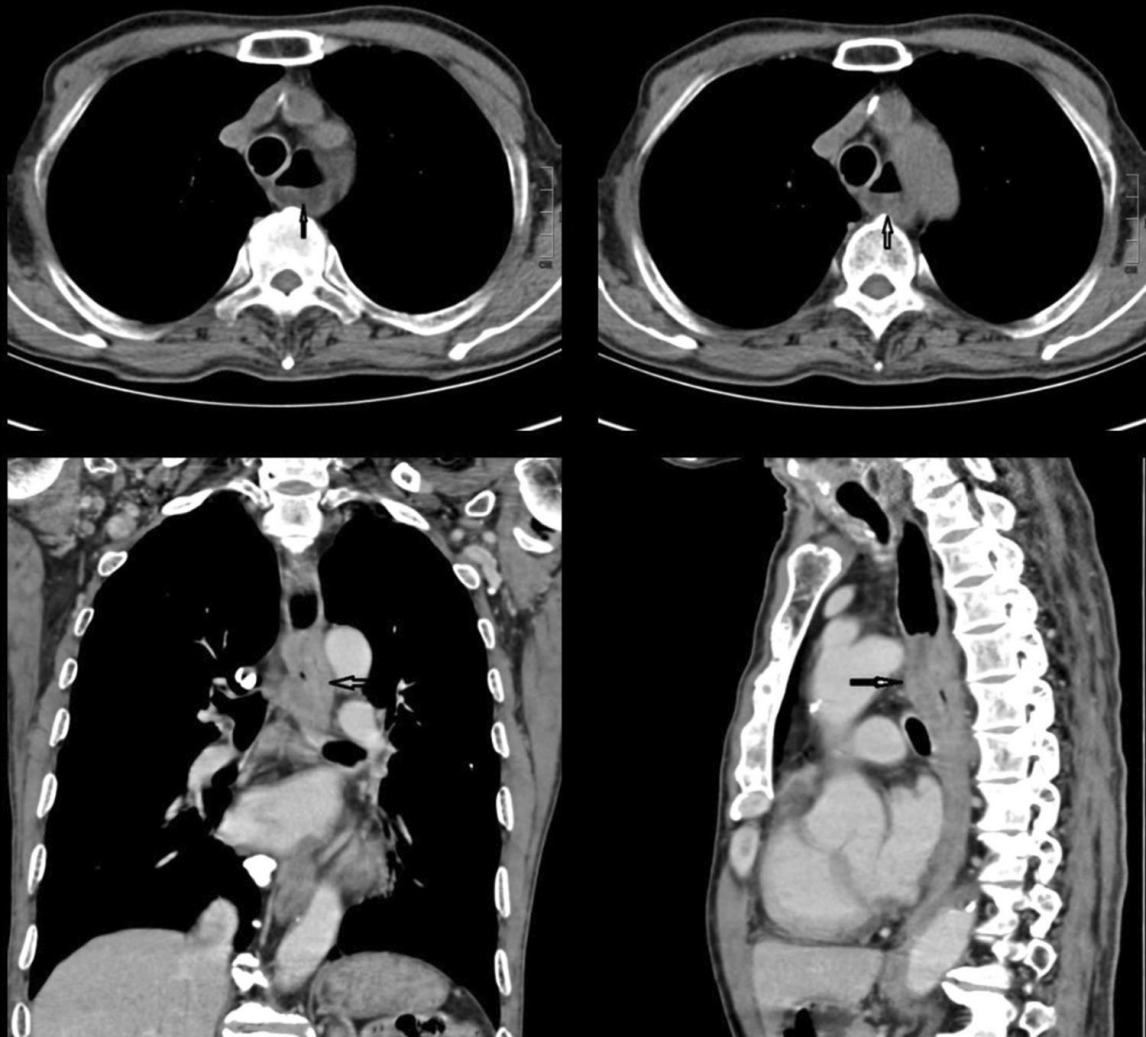
Control group computed tomography (CT) images. (**A**, **B**) Axial CT images showing the thickness of the esophageal wall, but the thickness could not be accurately measured because the esophageal lumen was not filled with contrast agents, and no ulcer was observed. **C** Coronal CT image of the blank control group, showing esophageal wall thickening, but esophageal thickness could not be measured because the esophageal lumen was not filled with contrast agent. **D** CT sagittal image showing thickening of the esophageal wall, but the thickness of the esophageal wall could not be measured because the esophageal lumen was not filled with a contrast agent, and no ulcer was observed

Figures 2, 3, 4, 5 show typical CT images for the four contrast agents. The esophageal filling degree was good and could meet the diagnostic requirements in all patients of the yogurt and lotus root powder. Nevertheless, since the scanning timing in the gas-producing powder group was difficult to synchronize perfectly with the agent intake, three participants (9%) had a poor filling degree and could not meet the diagnostic requirements. There were no significant differences in esophageal filling degree among the yogurt, lotus root powder, and gas-producing powder groups (*P* = 0.093) (Table 2). The consistency between the two observers was good (κ = 0.81).

**Table 2:** Score of esophageal filling degree of each group with oral contrast agent

Esophageal filling degree, *n* (%)	Yogurt group (*n* = 34)	Lotus root powder group (*n* = 34)	Gas-producing powder group (*n* = 34)	*P*
1 point	0	0	3 (8.8)	0.093
2 points	3 (8.8)	5 (14.7)	4 (11.8)	
3 points	29 (85.3)	28 (63.2)	22 (64.7)	
4 points	2 (5.9)	1 (2.9)	5 (14.7)	

### Concordance between CT with different oral contrast agents and pathological results

The CT results of 30 participants in the yogurt group were confirmed by the final pathological results, and three cases were false negatives. In the lotus root powder group, the CT results of 30 participants were consistent with the pathological results, with 3 false negatives. The CT results of 28 participants in the gas-producing powder group were consistent with the pathological results, with 5 false negatives. In the control group, the CT and pathological results were consistent in 25 participants, and 8 were false negatives. There was one false positive in each group. Table 3 presents the diagnostic values of CT when using one of the four contrast methods. The concordance rates were the highest for the yogurt (88.2%, with 91.7% specificity and 86.4% sensitivity) and lotus root powder (88.2%, with 92.3% specificity and 85.7% sensitivity) groups and the lowest for the control group (73.5%, with 90.0% specificity and 66.7% sensitivity).

**Table 3:** Comparison of CT examination results of esophageal carcinoma and pathological results with different oral contrast agents

	Histopathology/CT scan				Concordance rate	Specificity	Sensitivity
	+ / +	±	−/ +	−/−			
Yogurt group	19	3	1	11	88.2	91.7	86.4
Lotus root powder group	18	3	1	12	88.2	92.3	85.7
Gas-producing powder group	14	5	1	14	82.4	93.3	73.6
Control group	16	8	1	9	73.5	90.0	66.7

## Discussion

CT can be used for staging esophageal carcinoma, but esophageal collapse will lead to artifacts and false negative/positive. Therefore, this preliminary clinical trial aimed to investigate the filling state of the esophagus with different oral contrast agents for the staging of esophageal cancer by CT. The results suggest that yogurt mixed with ioversol could fill and expand the esophagus with minimal preparation, displaying the structure of the esophageal lumen and wall thickness. Therefore, this mixture of yogurt and ioversol might be used as a positive contrast agent for esophageal CT. Similar results were observed for the lotus root powder mixed with ioversol, but its preparation was more arduous. Indeed, the viscosity of the lotus root solution depends on the ratio between powder and water and temperature (it should be maintained at 40 °C for optimal results). Therefore, using the lotus root powder solution is impractical in a clinical setting.

EUS is considered the gold standard for esophageal lesions. Still, it also has some disadvantages, such as the inability to traverse high-grade malignant strictures for adequate T staging and inter-operator variability [18]. Besides, EUS can only examine the lymph nodes near the esophagus [18]. Therefore, CT is necessary for some patients who cannot underdo EUS (e.g., esophageal stricture) and adequate staging, especially for patients with advanced disease and lymph node involvement [5, 8–10]. Nevertheless, the improper esophageal expansion will lead to artifacts and increase the risk of false-negative and false-positive results [13–15]. Filling the esophagus with a contrast agent is conducive to the correct T staging and can provide more valuable imaging signs [16]. The currently used esophageal contrast agents, including positive contrast agents and negative contrast agents, must meet the following conditions: (1) can fill the esophagus; (2) have good contrast; (3) not easy to produce artifacts caused by excessive density differences; (4) low price and convenient source; and (5) good taste, harmless to the human body, and good tolerance [19, 20].

Because the esophagus is located in the thoracic cavity, the esophageal lumen is under negative pressure and closed under normal circumstances. Due to the anatomical characteristics of the esophagus, the false-negative diagnosis rate of the control group was significantly higher than that of the other oral contrast agent groups because the patients in the control group only took ioversol mixed with water, which cannot expand the esophageal lumen. In this study, by comparing the filling state of the esophagus, the attachment time of the esophagus inner wall, and the detection rate of esophageal carcinoma after oral administration of different contrast agents, it was found that the detection rates in the oral positive contrast agent groups were significantly higher than in the control group. The tumor detection rate of the gas-producing powder group (air group) was lower than that of the yogurt group and lotus root powder group, probably because the timing of swallowing the gas-producing powder in relation to imaging was not easy to synchronize, resulting in poor esophageal filling.

Due to the non-adhesive nature of the ioversol water agent, the short retention time, and the poor wall-hanging property, it was challenging to observe the esophageal mucosal defect and small lesions, which could not meet the ideal diagnosis requirements. Lotus flour powder and yogurt solved this problem and could increase the attachment time to the esophagus. Although gas-producing powder could not solve this shortcoming, the filling degree of the esophagus by air was like that of lotus root powder and yogurt, and the difference was not statistically significant. In this study, there were 34 participants in the gas group, and 5 were false negatives. Simultaneously, due to the interface effect, the displayed esophageal wall thickness was thinner than usual. When observing the images, different window widths needed to be used to adapt to changes in soft tissue and gas, so the use of gas-producing powder is still subject to certain disadvantages. In addition, the gas production process of the direct oral gas-producing powder was fast, and some patients were prone to coughing, resulting in poor cooperation during the examination and also affecting the examination effect, as observed for gastric examinations using such powder [21–23]. There was no statistically significant difference in esophageal filling degree and esophageal carcinoma detection rate between the lotus root powder and yogurt groups (*P* > 0.05), but due to the cumbersome preparation process of the lotus root paste (such as high requirements for the temperature of the water, amount, ratio, stirring, etc.), the yogurt was easier to prepare and was readily accepted by the patients. It tasted good, required minimal preparation, and was low-cost, convenient, and practical, but it also showed the range and length of the esophageal lesions. Future studies should also consider the use of double-contrast [24].

There were 11 false-negative diagnoses in the yogurt, lotus root powder, and gas-producing powder groups in this study. It was because, at least in part, the middle and lower segments of the esophagus were usually more filled than the upper part, which resulted in three false-negative diagnoses (one in each experimental group). Five other false negatives were probably due to the inadequate timing of taking the gas-producing powder and imaging, and three false negatives were early esophageal cancers with non-obvious thickening of the esophageal wall (one case in the yogurt group and one in the lotus root powder group).

This preliminary clinical trial had some limitations. First, the sample size was relatively small, and additional studies with larger sample sizes are needed to validate the findings. Second, there was a possible selection bias, as the analyses could not be stratified based on staging and pathological types of esophageal cancer. Third, this was a single-center study, and the generalizability of the findings is unknown. Fourth, it was an imaging study to determine the best contrast agent for esophagus filling during CT. What happened with the patients after imaging was not examined in this study. Finally, no other imaging modalities were performed.

## Conclusions

In conclusion, this preliminary clinical trial suggested that yogurt mixed with ioversol could fill and expand the esophagus with minimal preparation, displaying the structure of the esophageal lumen and wall thickness. Yogurt mixed with ioversol might be used as a positive contrast agent for CT esophagography. Similar results were observed for the lotus root powder mixed with ioversol, but it required more preparation. Future studies are still necessary to confirm the results.

## Data Availability

Data are available upon reasonable request from corresponding author.
